# Long-Term Assessment of an Innovative Mangrove Rehabilitation Project: Case Study on Carey Island, Malaysia

**DOI:** 10.1155/2014/953830

**Published:** 2014-07-03

**Authors:** Shervin Motamedi, Roslan Hashim, Rozainah Zakaria, Ki-Il Song, Bakrin Sofawi

**Affiliations:** ^1^Department of Civil Engineering, Faculty of Engineering, University of Malaya, 50603 Kuala Lumpur, Malaysia; ^2^Institute of Ocean and Earth Sciences (IOES), University of Malaya, 50603 Kuala Lumpur, Malaysia; ^3^Institute of Biological Sciences, Faculty of Sciences, University of Malaya, 50603 Kuala Lumpur, Malaysia; ^4^Department of Civil Engineering, Inha University, 100 Inha-ro, Nam-gu, Incheon 402-751, Republic of Korea

## Abstract

Wave energy and storm surges threaten coastal ecology and nearshore infrastructures. Although coastal structures are conventionally constructed to dampen the wave energy, they introduce tremendous damage to the ecology of the coast. To minimize environmental impact, ecofriendly coastal protection schemes should be introduced. In this paper, we discuss an example of an innovative mangrove rehabilitation attempt to restore the endangered mangroves on Carey Island, Malaysia. A submerged detached breakwater system was constructed to dampen the energy of wave and trap the sediments behind the structure. Further, a large number of mangrove seedlings were planted using different techniques. Further, we assess the possibility of success for a future mangrove rehabilitation project at the site in the context of sedimentology, bathymetry, and hydrogeochemistry. The assessment showed an increase in the amount of silt and clay, and the seabed was noticeably elevated. The nutrient concentration, the pH value, and the salinity index demonstrate that the site is conducive in establishing mangrove seedlings. As a result, we conclude that the site is now ready for attempts to rehabilitate the lost mangrove forest.

## 1. Introduction

Storm waves and erosion damage coastal environments and hurricanes inundate and destroy coastal properties [1]. Storms also cause severe short-term erosion, that is, more intensive than that caused by seasonal fluctuations [2]. Coastal zones need proper protection from these threats. Shoreline protection techniques should in fact prevent potential damages and restore erosion imbalances originating from natural or anthropogenic causes [3]. An understanding of effective protective mechanisms and their implementation is essential for maintaining and preserving natural elements near the shores [4–7].

Human interference has a direct and consistent influence on coasts [8, 9]. Natural resources can be lost when artificial structures are installed. Steel and concrete have been used to construct coastal structures without considering the ecological imbalances that they might cause [10, 11]. Artificialization near the coast, then, should be minimized to the extent possible.

Rigid one-directional approaches cannot fully encapsulate the needs of prediction of coastal systems [12–15]. Therefore, rigid approaches should be coupled with ecological engineering to deal with the consistent loss of ecosystem elements caused by artificialization. The concept of ecological engineering was introduced 30 years ago [16]. The concept of incorporating ecoengineering with coastal protection, however, has developed more recently.

The loss of mangrove forests in the last two decades is a unique example of human intervention in a coastal system [9, 17]. Mangrove forests have been destroyed for industrial and agricultural purposes, timber and charcoal production, and shrimp farming. If the current decline rate persists, mangrove forests will vanish entirely in the next 100 years [18]. For example, Sungai Haji Durani in Selangor, Malaysia, suffered from excessive mangrove losses before ecocoastal protection was put in place. With the help of ecotechnologies, the site has been restored to a significant shelter for fauna and flora [19, 20].

In this paper, we briefly review the global threats to mangrove forests. Next, we discuss the potency of reestablishment for a dominant mangrove species (*Avicennia marina*) from engineering perspective. Finally, we introduce an innovative shoreline protection scheme for Carey Island in Selangor, Malaysia, in which a breakwater system is integrated with mangrove rehabilitation. We used various postassessment methods such as sedimentologic characterization of the coast, cross-section profiling, hydrogeochemical analysis of the soil and water, and cost estimation to characterize the shoreline of Carey Island for future mangrove rehabilitation projects.

## 2. Threats to Mangrove Forests

Mangroves are defined as individual plants in a mangrove forest community [21]. Ghazali [22] defined mangroves as plants existing in muddy, high-moisture soils of tropical and subtropical tidal waters. Mangrove forests grow only in accumulative forests situated at the verge of land and sea in tropical and subtropical latitudes between 25°N and 25°S [23]. They also grow naturally in sheltered coastal zones and on islands with locally variable topography and hydrology [24]. The forests consist of wide and unique varieties of vegetation that can grow despite exposure to wave impacts and water salinity in the harsh coastal environment.

The importance of mangroves is well documented in the literature [25–27]. Mangroves are renowned for their high biological productivity and nutrient source. For example, mangroves provide a suitable environment for breeding and serve as a nursery ground for marine species such as fish and terrestrial vertebrates [28]. In addition, the mangrove roots are capable of absorbing wave energy, which means that they can stabilize shoreline erosion and act as a natural barrier to protect the shoreline from devastating wave impacts generated by tsunamis and other storms [29]. For example, Duarte et al. [30] stated that tall mangrove trees are capable of significantly reducing wave energy.

A recent study estimated that the total mangrove forest area in 2005 was 137,760 km^2^ in 118 countries and territories in tropical and subtropical zones [23]. The authors stated that the largest extent of mangrove forest is found in Asia (42%), followed by Africa (20%), North and Central America (15%), Oceania (12%), and South America (11%). Alongi [31] wrote that the most diverse biogeographical mangrove forests are in the Indo-Pacific region with Indonesia, Australia, Brazil, and Nigeria.

Recently, rapid growth in population, industrialization, and urbanization has diminished the mangrove forests [30]. Based on the most recent assessment which assessed the trends of mangrove loss between 1980 and 2005, the global existing forests have disappeared at annual rate of over 2% [32]. For example, in Malaysia, Ghazali [22] claimed that approximately 160 km^2^ of mangrove forests vanished each year from 1980 to 2001. As a result, mangrove restoration project is now an outstanding issue for shoreline protection.

### 2.1. * Avicennia Marina*: A Representative Mangrove Species in Peninsular Malaysia

Most of the western coastline in Peninsular Malaysia is fringed with mangrove forests in mud flats. The island of Sumatra in Indonesia shelters the seas in the Strait of Malacca, bringing a relative calmness to the seas in the western peninsula compared to eastern Malaysia, which faces the South China Sea. Alluvial plains are a common feature of the coasts on the western peninsula [29]. The rivers discharging to the straits on the west coasts carry mostly fine silt and clay that contribute to alluvial plain formation. The clayey deposits on the western coast of Malaysia provide a proper substrate for* Avicennia marina* mangroves, that is, conducive to their viability [20]. Therefore, the pioneer species of mangrove in Peninsular Malaysia is* Avicennia marina,* which propagates through seeding [22].

If the waters are relatively calm and the geotechnical stratum is well elevated with respect to mean sea level (MSL),* Avicennia marina* can take root [33]. When* Avicennia marina *forests grow to a certain height, the forests attenuate waves [34]. Wave attenuation leads to faster sediment deposition behind the mangrove forests [32, 35]. Increases in the levels of sediments behind the trees block the ebb flow of the tide [36, 37]. As a natural pond is forming, rainwater and the fresh water coming from upstream decrease the salinity of the pond.* Avicennia marina* cannot survive in a constant inundation of diluted saline water [33] and eventually dies. In addition, new seeds cannot grow in the target area because they cannot reach the ground, remaining afloat in the water. This process can be accelerated by the significant rise of the MSL.

## 3. Materials and Methods

Carey Island is located in the Banting district in Selangor, Malaysia. A mangrove replantation project was performed in the target area between 2008 and 2010. Evaluating the success of mangrove rehabilitation requires three to five years [19], so it is now time to evaluate the project. In the case study described here, we assess the current condition of Carey Island in terms of sedimentologic characteristics, hydrogeochemical aspects of soil and water, and topographical features of the associated beaches. We also assess the feasibility of future mangrove rehabilitation projects at the target site.

### 3.1. Description of Site at Carey Island, Malaysia

#### 3.1.1. Geographical Location

**Figure 1:**
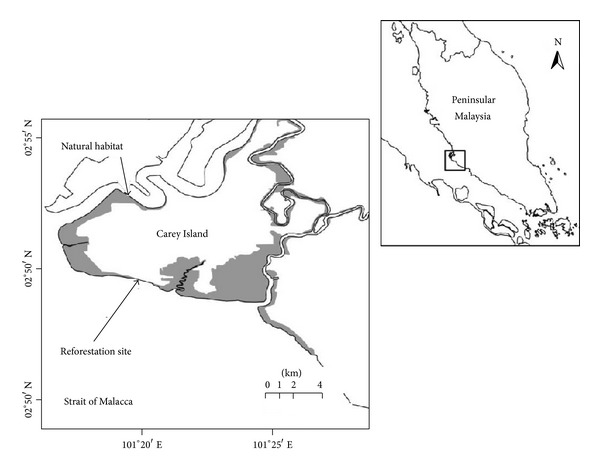
The geographical position of Carey Island. Gray-shaded hatch denotes the thin mangrove cover.

Carey Island is located within the Klang Isle (03°38′N and 101°00′E), which is one of the most famous mangrove forest reserves in the Strait of Malacca alongside the west coast of the Malaysia Peninsula (see Figure 1). Klang Isle is composed of eight small islets, and Carey Island is the largest of the islets, separated from the Selangor coast by the Langat River on the east and the Klang River on the north. The total area of Carey Island is 161.87 km^2^ and nearly 65% of its total area is covered with palm oil trees [38]. The island's elevation is lower than the mean high tide level. Consequently, to protect the upper land from the effect of higher waves, coastal dikes, a network of drainage canals, and water control systems have been constructed [26].

#### 3.1.2. Geology and Sediment Stratigraphy

Carey Island is located next to the wider channel of the Strait of Malacca. Previous geological studies at Carey Island demonstrated the existence of alluvial textures [39–41]. Bathymetry studies in the Strait of Malacca reveal complicated characteristics, and the area has been interpreted as a “Pleistocene lowered-sea-level alluvial-delta-fan” system [42].

Approximately 70% of Carey Island is composed of Holocene deposits of clay, silty clay, peat, and minor sand formations that overplaced on Pleistocene deposits of gravel, sand, clay, and silt [38, 40]. Sedimentary rocks comprise the underlying bedrock at the site. The bedrock is similar to that found in Selangor, Malaysia. Therefore, the bedrock at the Carey Island mainly constituted of interbedded shale, siltstone, and sandstone [43].

#### 3.1.3. Climatic Properties

Tropical climate characteristics are widely found within the target site, and the climatic properties in this zone are tied to northeast and southwest monsoonal flows. During the intermonsoon season, the weather is highly unpredictable [20].

The direction of wind alters in accordance with the monsoon seasons. Based on a report from the Malaysian Meteorological Department (MMD) in 2009, prevailing wind direction, measured in the range from lat 2.0°N to 3.0N and long 101.0°E to 102.0°E, was 160°SSE with an average velocity of 2 m/s. These winds generate waves with heights of 0.1 to 1.5 m on the coasts of Carey Island with wave periods of 2~8 s [44].

In terms of temperature, humidity, and annual precipitation, MMD reported that the temperature at the site has fluctuated between 35°C and 26°C with an average humidity of 92% since 2000. In addition, rainfall data indicate that, on average, the total annual precipitation at the site has been 2.225 m for the last ten years [44].

### 3.2. Rehabilitation Method Using Ecoengineering Coastal Protection

**Figure 2:**
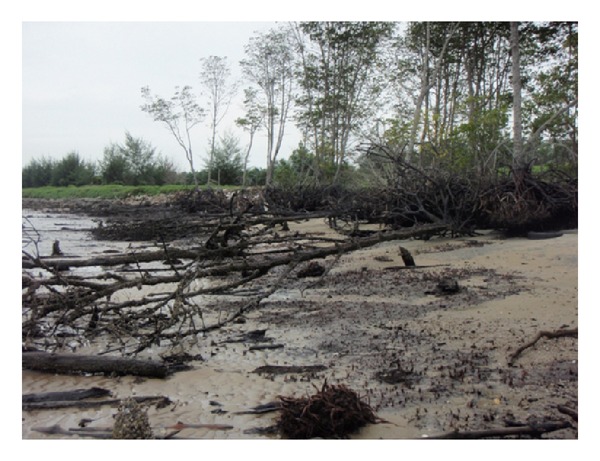
Loss of “*Rhizophora apiculata*” mangrove forests at the site before the project is carried out in early 2008.

The clayey beach of Carey Island has been a natural residence for mangrove trees for centuries. Since 1950, large areas of mangrove forest have been converted to agricultural areas based on long-term national plans. During the 1970s, a coastal line-stretched dike was erected to shelter the agricultural land in the higher area of the Carey Island coast. Department of Irrigation and Drainage (DID), Malaysia, built hundreds of kilometers of these coastal dikes nationwide to protect agricultural lands from tidal inundation and massive wave energy induced by storms [20]. The environmental value of mangrove forests, however, was ignored in this era, and the negative consequence was the massive retreat of mangrove trees observed over the last two decades (Figure 2).

The dikes behind the mangrove forests can enclose water, creating ponds between the mangroves and the dikes [19]. In addition, the level of the silty-clay layer on the beachfront has decreased, which led to scouring of the soil underneath the mangroves [20]. This, in turn, caused depletion of the mangroves.

The first mangrove rehabilitation project, which was funded by Sime Darby Plantation Sdn Bhd, was carried out on the southern part of Carey Island for three years between 2008 and 2010. That part of Carey Island, which faces the Strait of Malacca, was chosen for rehabilitation because of serious erosion and sustained forest degradation. In 2008, the University of Malaya conducted a pilot research project involving an innovative shoreline protection scheme to rehabilitate depleted mangrove forests on Carey Island. In this project, a coastal defense structure was integrated with ecological engineering methods.

**Figure 3:**
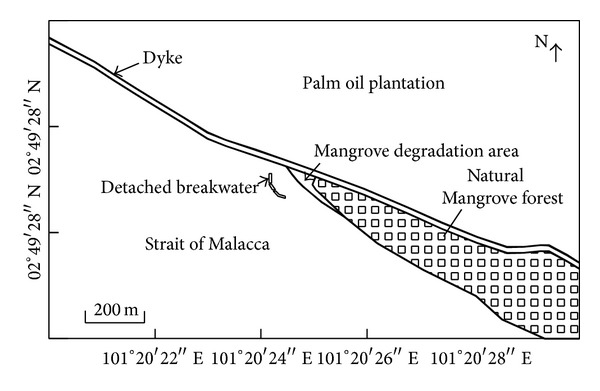
The schematic view of Carey Island after mangrove rehabilitation project in 2008.

The university directed construction of a detached breakwater system composed of a submerged breakwater with three segments separated by 2.5 m to allow for water circulation. Hashim et al. [20] reported that the average crest height of the breakwater system is 1.8 m above MSL. Furthermore, the system occupies nearly 60 m of the shore at the site. The system is approximately perpendicular to the coastline. The authors also presented detailed information on the breakwater design and described its implementation.  Figure 3 illustrates the geographic position of the deforested area and the existing mangrove forest before the breakwater system was constructed in 2008. The figure also depicts the breakwater system and the dike after construction was completed.

Once the breakwater structure was finished in early 2009, mangroves were replanted. Nursery-raised 20-cm-tall seedlings of* Rhizophora apiculata* and* Avicennia marina* were chosen because* Avicennia marina* is the most dominant species on Carey Island and* Rhizophora apiculata* is the major species at the target site [45]. Both species were planted in a grid system using coir logs and conventional planting methods. Almost all the planted seedlings died within one year because of toppling by high tides and waves (survivability index = 5%).

**Figure 4:**
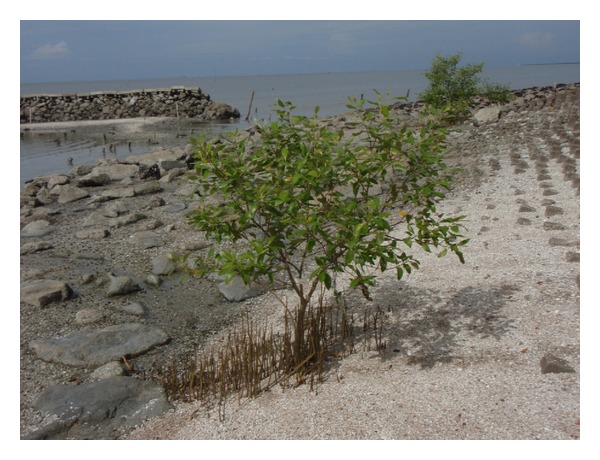
A row of natural recruits in the study site at the site near the detached breakwater (June 2014).

Our most recent observation (June 2014) revealed a few new natural recruits outside the rehabilitation site. The natural recruits are* Avicennia marina* and* Rhizophora apiculata*, which are now more than 1 m tall with well-developed spreading root systems (Figure 4). The growth of natural recruits indicates that the site is now biologically ready for further mangrove rehabilitation. Based on our findings, trying to rehabilitate the mangrove forest at the target site is worthwhile.

### 3.3. Characterization of the Carey Island Shoreline

#### 3.3.1. Sedimentologic Characterization

For this study, a stainless steel soil sampler was used to collect 36 soil samples from the Carey Island shoreline. The soil sampling was carried out in accordance with ASTM International's* Standard Practice for Field Collection of Soil Samples for Subsequent Lead Determination* (ASTM E 1727). We used sieving and hydrometer methods to obtain the particle size distribution of the soil samples. The tests were performed in accordance with the* Standard Test Method for Particle-Size Analysis of Soils* (ASTM D 422). According to the prerequisite criteria of ASTM D 422, this test is applicable only to sediment including fine sand, silt, and clay particles that are larger than 0.075 mm. Before carrying out the test, we removed carbonates, soluble salts, organic matter, and iron oxides from the samples. For all samples, the soil mass was dried and sieved.

#### 3.3.2. Cross-Section Surface Profiling

**Figure 5:**
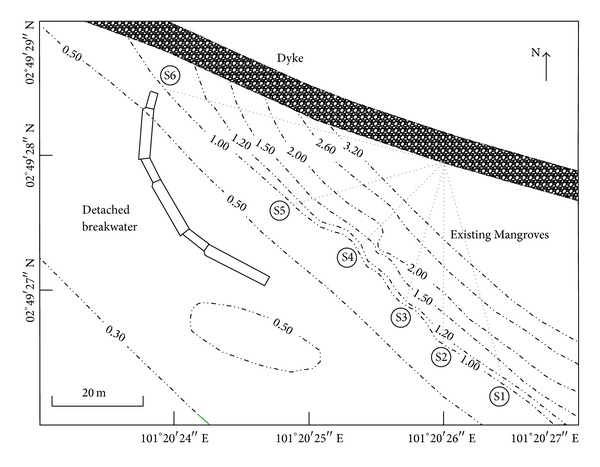
Thirty-six soil samples have been collected along S1 to S6 lines in various topographic elevations.

The bathymetric data was collected using a TOPCON Total Station. The Temporary Bench Mark (TBM) was located at 2°49′28′′N, 101°20′25′′E. Further, surface profiling was conducted in accordance with TBM along axis S5 in Figure 5 from 0 to 60 m of the existing dyke with the interval of 5 m. Finally, the surveyed data points were corrected in relation to the coordinates taken from TBM [20].

We collected bathymetric data from Carey Island three times: in (1) December 2008 (before the detached breakwater system was constructed); (2) January 2010 (12 months after the detached breakwater system was complete); (3) January 2013 (48 months after the detached breakwater system was finished). The analysis of bathymetric data along the S5 axis in Figure 5 shows the variation of average elevation of a cross-section of the beach.

#### 3.3.3. Hydrogeochemical Assessment

We performed an extensive study on the hydrogeochemical properties of soil and water samples taken from Carey Island's coastal zone. In all, we collected 36 water samples along axis S1 through axis S6 in Figure 5. Along each axis, six samples were taken at the longitudinal distance of 10 m. The S1, S2, and S3 axes denote the area of existing mangroves (natural habitat) and the S4, S5, and S6 axes represent the restoration site. The natural habitat can be considered as the control site and the restoration site can be considered as the experimental site. The pH value, salinity index, nutrient concentration, and heavy metal content of the samples were measured.

We assessed the pH value for each of the 36 water samples using a multiprobe apparatus according to the* Standard Test Methods for pH of Water* (ASTM D 1293) [46].

Sample salinity was assessed using an Atago Hand-Held Refractometer in accordance with* Standard Methods for the Examination of Water and Wastewater* (American Water Pollution Control Federation) [47]. Figure 5 shows the location of collected water samples for salinity test aligned along measurement axes S1 through S6.

The nutrient concentrations were measured via ICP (the 861-Advanced Compact; Australia/Switzerland) in accordance with ASTM Water Testing Standards in the “Inorganic Constituents in Water” series [48, 49]. In this study, we evaluated the concentration of* nitrogen*,* sulfur*,* chlorine*,* calcium*,* manganese,* and* copper*. The locations of collected water samples are depicted in Figure 5.

## 4. Results and Discussion

### 4.1. Sedimentology

Based on the soil analysis results, we found that sample consisted of 76.14% of silt and clay (on average) and only about 23.86% of fine sand. The mean grain size of the soil particle was 0.016 mm. Most of studies done on Carey Island confirm these results, reporting that silt and clay were the dominant types of soil found on the island, ranging from 50% to 55% of total substrate soil composition [33, 45]. Although mangroves can grow in sandy soils, they prefer to take root in fine clay and alluvial soil [20, 24]. We observed a considerable increase in the amount of silt and clay constituents in this study.

An important part of a mangrove restoration project is finding a site with the most suitable conditions for mangrove establishment [31]. The existing remnants of mangroves in the Carey Island mud flat testify that the mud flat was once sedimentologically rich enough to retain mangrove roots. Therefore, restoring clay and silt to the site may result in more stable sedimentologic conditions that will help mangroves grow faster. We can conclude that the site is now sedimentologically ready for undertaking mangrove restoration projects.

### 4.2. Cross-Section Surface Profiling

**Figure 6:**
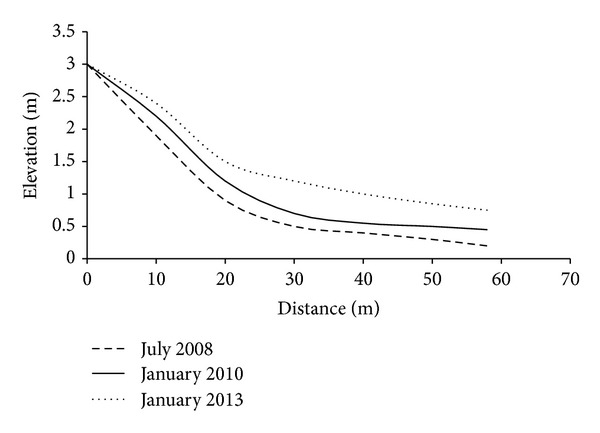
Observed elevation of sea bed level from December 2008 until January 2013 at the site.

Figure 6 shows the variation of elevation from July 2008 to January 2013. About four years into the project, the elevation of a cross-section along axis S5 in Figure 5 had increased considerably. For example, one month after construction, the elevation at the toe of the breakwater system rose from 0.2 m to 0.45 m. After 12 months, it increased nearly 125%. In the same way, the data collected in 2008 indicate that the elevation of the soil stratum at a distance of 30 m from the shoreline was about 0.5 m, and as of January 2013, it had increased to 1.2 m. The elevation was about 1.9 m near the coastal dike at a distance of 10 m from the shoreline in 2008. The elevation at the same location reached 2.2 m in January 2010. Finally, in January 2013, the elevation leveled at 2.4 m. The sediment deposition trend is more noticeable near the breakwater than near the shoreline. Based on the positive trend of growth in the seabed level over time, we can conclude that the elevation of a cross section of the beach is increasing.

Detached breakwater systems are constructed not only to dampen the energy of waves to create a calm hydraulic environment in the nearshore zone but also to positively alter sediment deposition trends for compensation of transported littoral sediments [20]. Consequently, measuring sediment deposition elevation and collecting bathymetric data from near shore are essential to ensuring the success of any coastal protection project.

Generating a sedimentologic environment, that is, conducive to the establishment of mangroves, is crucial in ecological restoration projects [19]. The positive trend in sediment accumulation in the nearshore area indicates sedimentologic success after segmented breakwater systems have been constructed [20]. We can conclude that the future establishment of mangroves would be successful at the target site based on the suitable seabed level.

### 4.3. Hydrogeochemical Evaluation

#### 4.3.1. pH Value

The results show that the pH values in the soil water did not differ significantly between the reforestation site and the natural habitat. The average and standard deviation of pH value for the reforestation site is 7.23 ± 0.19; for the natural habitat, pH is 7.14 ± 0.21. This indicates that the pH values at both sites are slightly alkaline. Because mangroves can survive in neutral to high pH conditions [50], the pH level at this site is appropriate for planting mangroves.

The pH value is important in determining the chemical properties of water. Lower pH values (<7) increase the metal availability in the medium because the hydrogen ion (H^+^) has a higher affinity for attracting negative charges and releasing the metals to the environment. Conversely, higher pH values (>7) decrease the metal availability of the soil because the hydroxyl ion (OH^−^) has high potential to attract positive charges and lower the availability of metals in the soil and water [51]. The pH values measured, then, show that the water samples are slightly alkaline. The slight alkalinity of samples can reduce the concentration of heavy metal ions along the observed coastal zone.

#### 4.3.2. Salinity Index

The mean value of salinity for the reforestation site ranged between 24.3 ± 0.43 ppt and 29.2 ± 0.12 ppt (average = 26.9 ± 0.15). For the natural habitat, the values ranged from 25.7 ± 0.57 ppt to 28.3 ± 0.74 ppt (average = 26.3 ± 0.62 ppt). Therefore, the results show that the salinity in the water samples did not vary significantly between the reforestation site and the natural habitat.

Salinity plays an important role in determination of water and soil properties. Salinity is dominantly affected by bathymetric properties [52]. At lower sea levels, the salinity is naturally maintained between 33 ppt (part per thousand) and 38 ppt; in highly elevated zones, the salinity might vary between 1 ppt and 25 ppt [53]. In addition, Joshi and Ghose [54] stated that the salinity of a medium decreases as the distance from the coastline increases. Therefore, the lower the elevation of a seabed, the higher the salinity.

Mangroves are well known for their halophyte characteristics [55], which allow them to survive in high salinity through certain mechanisms of salt tolerance [50]. This explains the dense mangrove community in the seaward fringe and riverine estuaries where the salinity is high. For example, the seaward mangrove species (i.e.,* Avicennia marina*) has evolved over the years to be more flexible in high salinity and pH environments [52].

Salinity greatly affects the growth of plants. Various studies have been conducted to examine the survival of mangroves in different salinity ranges [56–59].* Avicennia marina* is always recognized as the one of the most salt-tolerant mangrove species because it naturally grows on the waterfront [56, 58]. Conversely, many other mangrove species cannot survive on the seafront because they cannot tolerate higher water salinity [52, 59]. Clearly, selecting the proper mangrove species for the local conditions is crucial for successful restoration projects.

The same mangrove species could have different tolerances to salinity depending on the region. Aziz and Khan [52] discovered that, in India,* Avicennia marina* can survive in semiarid to saline deserts and is highly salt-tolerant up to 35 ppt. However, it has been found that* Avicennia marina* in Hong Kong can barely survive in salinity higher than 15 ppt [60]. In Malaysian coastal zones, mangroves can survive in salinity between 20 ppt and 30 ppt [33]. We found that the salinity ranges from 24.3 ± 0.43 to 29.2 ± 0.12 ppt (average = 26.9 ± 0.15 ppt) on Carey Island. We can conclude, then, that the target site is suitable for natural recruits of* Avicennia marina*.

#### 4.3.3. Nutrient Concentrations

During palm cultivation on Carey Island, the use of fertilizer is inevitable. The target site of this study is near three rivers: Air Hitam, Judah, and Keluang [40]. The associated surface water runoff can bring fertilizer remnants into the rehabilitation site because the site is at the edge of the palm plantation areas. In addition, the growth of mangroves is more noticeable near riverine locations and fringe areas where the nutrients can be transported [61]. Because mangrove seedlings could use the abundant nutrients in the sediment to establish themselves, quantifying the nutrient content at the site is worthwhile.

Nutrients are essential chemical components required for plant growth and life-cycle completion. Nutrient-rich sediments quicken the growth of mangrove seedlings [61]. In this section, we quantitatively assess nonorganic nutrients in the soil and water samples. Nonorganic nutrients can be separated into two major groups: macronutrients and micronutrients. Plants usually require large proportions of macronutrients to grow (occurrence of internal chemomalicious activities) [62]. On the other hand, micronutrients are required in smaller amounts to be involved in catalytic and regulatory mechanisms [60]. If the micronutrients exceed an acceptable level, they can toxify plants [51]. In such conditions, the micronutrients are also called “heavy metals” [51].  Table 1 presents nutrient concentrations, which are discussed in detail in the sections that follow.

Nitrogen is a vital element for the growth of plants. Nitrogen in soil can be found in either organic or inorganic form. The organic nitrogen in soil is composed of small plant residues, small living organisms, and decomposing organic matter. The inorganic form is nitrate (NO_3_^−^) or ammonium (NO_4_^+^) [63]. According to Table 1, the nitrate concentrations at the reforestation site were recorded in the range from 22.69 ± 0.19 ppm to 57.39 ± 0.33 ppm (average = 42.1 ± 0.27 ppm). The allowable range of nitrogen in the soil is between 10 ppm to 60 ppm [55], so nitrogen is in the normal range at the reforestation site. Conversely, no nitrate was found at the natural habitat. This might be related to the consumption of nitrogen through processes such as bacterial nitrification, ammonia valorization, and denitrification [63]. We can conclude that the reforestation site has enough nitrogen for mangrove establishment.

Sulfur is the essential chemical component of the structure of plants and biological processes. Plants use sulfur in the form of sulfate (SO_4_^2−^) [62]. The observed value of sulfate is almost the same at the reforestation site (1.56 ± 0.38 ppm to 4.01 ± 0.12 ppm, average = 2.71 ± 0.51 ppm) and the natural habitat (1.78 ± 0.58 ppm to 4.39 ± 0.86 ppm, average = 2.73 ± 0.62 ppm). The minimum value for sulfate in the coastal area must not be less than 2 ppm [64]. There is sufficient sulfate at both sites, and the reforestation site has enough sulfate for mangrove establishment.

Chlorine is an important chemical element for photosynthetic reactions in plants [62]. Plants use chlorine in the form of chloride (Cl^−^), a highly abundant and soluble element in the environment. The chloride concentration at the reforestation site was higher (15.46 ± 0.41 – 32.84 ± 0.76 ppm, average = 26.76 ± 0.84 ppm) than that at the natural habitat 12.08 ± 0.37 – 27.91 ± 0.54 ppm, average = 19.32 ± 0.41 ppm). Generally, the mangroves can endure chloride salts up to 106 ppm [63], so the amount of detected chloride in the study area cannot be regarded as the cause of mangrove degradation. The chloride content at the reforestation site is within the allowable range.

Calcium and magnesium are important nutrients for plant growth [63, 65]. The concentrations of calcium (260.28 ± 0.79 – 314.81 ± 0.11 ppm, average = 281.16 ± 0.36 ppm) and magnesium (265.47 ± 0.94 – 325.71 ± 0.21 ppm, average = 291.23 ± 0.36 ppm) at the reforestation site were reasonably similar to the natural habitat concentrations of calcium (151.60 ± 0.16 ppm – 311.47 ± 0.52 ppm, average = 273.19 ± 0.48 ppm) and magnesium (209.00 ± 0.49 ppm – 255.77 ± 0.87 ppm, average = 283.34 ± 0.36 ppm). Clemens [66] claimed that calcium and magnesium should be classified as noncritical metals if the calcium amount is below 500 ppm and the magnesium content is below 300 ppm. The obtained concentrations of both nutrients can be considered normal, meaning that the reforestation site is an appropriate ground for mangrove establishment.

Manganese acts as an activator of several enzymes in plants responsible for the photosynthesis, respiration, and synthesis of proteins [64, 67, 68]. The manganese content should be in the range between 0.01 ppm and 0.8 ppm [69]. The manganese concentration at the reforestation site (0.09 ± 0.29 – 0.78 ± 0.74 ppm, average = 0.36 ± 0.31 ppm) is a little higher than that of the natural habitat (0.01 ± 0.06 – 0.38 ± 0.24 ppm, average = 0.29 ± 0.44 ppm). The concentrations of manganese at both sites are considered favorable for mangrove growth.

Copper is classified as a metal that is extremely toxic to plants (Reddy and D'Angelo 1997) [64]. The presence of excessive copper content in the soil and water near plants leads to chlorosis, yellow coloration, and retardation of plant growth [62]. The concentration of copper is slightly higher at the natural habitat (0.02 ± 0.53 – 0.04 ± 0.17 ppm, average = 0.037 ± 0.31 ppm) than at the reforestation area (0.01 ± 0.63 – 0.02 ± 0.27 ppm, average = 0.031 ± 0.68 ppm). The average allowable value of copper in plants and soil is between 0.02 ppm and 0.05 ppm [69]. The current concentration of copper at both sites is considered normal for plant growth.

In summary, we can conclude that the reforestation site is suitable for further attempts to rehabilitate the lost mangroves because the concentrations of nutrients are favorable for their growth. Future mangrove rehabilitation trials can be successful with a slight alteration in wave energy reduction, entrapment of sediments to elevate the geotechnical datum, and rigorous anthropogenic control.

### 4.4. Cost Estimation for the Mangrove Rehabilitation Project

It is difficult to estimate costs for the mangrove rehabilitation projects in detail. Because the allocated budgets for the projects are relatively higher than the actual costs, a detailed cost estimation would help reduce unnecessary budgeting and might prevent inaccurate financial estimations [70, 71]. Based on the available project documents, we present a detailed cost estimation of the mangrove rehabilitation project integrated with the coastal protection system in this section.

According to the Department of Statistics Malaysia, the average inflation rate was about 2.66% between 2005 and 2013.  Table 2 presents the cost estimation for the full length of a structural part of the project including an 80-m-long and 2.5-m-wide detached breakwater system. The construction of the detached breakwater system for 1,920 m^2^ (the total occupied area of detached breakwater system) costs USD 23,154. Considering the inflation rate of 2.66% over the four years following construction, construction costs reached USD 25,910 in 2013. The financial estimates in mangrove restoration projects are reported for 0.01 km^2^ rather than for the area of the study site. As a result, the total construction cost for the detached breakwater system was USD 121,000 per 0.01 km^2^, which reached USD 135 thousand per 0.01 km^2^ considering the inflation rate over five years.

Table 3 presents the detailed cost estimation for the mangrove replantation. Between April 2009 and April 2010, 679* Avicennia marina* and 351* Rhizophora apiculata* mangroves were planted at the rehabilitation site. The total cost of mangrove replantation for 1,920 m^2^ was USD 3,366. After considering the inflation rate, it would be USD 3,814 in present time. At the time of the project, mangrove replantation at the site cost USD 175,000 per 0.01 km^2^. After taking the inflation rate into account, it would be USD 199,000 per 0.01 km^2^ in present time. These numbers imply that at a large scale, mangrove replantation is more costly than construction of coastal protection structures.

In conclusion, the total budget of the project, including the detached breakwater construction and the mangrove replantation, was about USD 296,000 per 0.01 km^2^ in 2008. Considering the inflation rate, it would cost USD 336,000 per 0.01 km^2^ in 2013. The aim of this part of our study was to introduce the detailed cost estimation of the project based on the available documents. The process of cost estimation can serve as a good reference for future projects.

## 5. Conclusion

In this paper, we provided a history of coastal protection practices in Malaysia since 1950. We also presented an example of an innovative mangrove restoration project on Carey Island, and investigated the feasibility of success for future mangrove rehabilitation projects at the site. Four years after the construction of the breakwater system, the sedimentologic characterization and bathymetric properties of the site have been enhanced. We found that silt and clay content had increased at the site up to 76.14% (on average) of collected samples and only about 23.86% of fine sand. The bathymetric data show an increase in seabed elevation after four years into the project. Based on the positive trend of growth in the seabed level over time, we can conclude that the elevation of a cross section of the beach is increasing. The pH value, salinity index, nutrient concentration, and heavy metal content of the samples were measured. In addition, according to the hydrogeochemical assessment, the site is conducive to establishing mangroves. Based on our findings, trying to rehabilitate the mangrove forest at the target site is worthwhile. For successful restoration, however, future research must focus on selecting mangrove species that can live in harmony with existing vegetation.

## Figures and Tables

**Table 1:** The range of nutrient concentration at reforestation site.

Nutrient concentration (ppm)	Reforestation site (ppm)	Natural habitat (ppm)	Concentration considered toxic (ppm)
Macronutrient			
Nitrogen (N)	22.69–57.39	0	10–60^a^
Calcium (Ca)	260.28–314.81	151.60–311.47	<500^b^
Magnesium (Mg)	265.47–325.71	209.00–255.77	<300^b^
Sulphur (S)	1.56–4.01	1.78–4.39	>2^c^
Micronutrients			
Manganese (Mn)	0.09–0.78	0.01–0.38	0.01–0.8^c^
Chlorine (Cl)	15.46–32.84	12.08–27.91	120–300^d^
Copper (Cu)	0.01-0.02	0.02–0.04	0.02–0.05^d^

Note: ^a^Gong and Ong [55], ^b^Clemens [66], ^c^Reddy and DeLaune [69] and ^d^Hopkins and Hüner [62].

**Table 2:** Estimation of hard-engineering restoration of the project at Carey Island in 2008 and 2012 with consideration of inflation rate.

No.	Item	Description	Unit	Unit cost (RM)	Total quantity for 80 m length	Total cost 2013 (RM) with inflation rate	Total cost 2008 (RM)	Total cost 2013 (USD) with inflation rate	Total cost 2008 (USD)
1	L-block interlocking (60 × 75 cm), concrete: grade 45, steel bar (diameter 10)	Ready mix concrete (normal mix), grade 45	m^3^	193	27.5288	5,313.06	4,747.75	1,736.29	1,551.51
		Steel bar	ton	2216.67	1.69612	3,759.74	3,359.70	1,228.67	1,097.94

2	Stone (D50 = 17–20 cm)	density: 2300 kg/m^3^	ton	30	637.63	19,128.90	17,093.59	6,251.27	5,586.14

3	Gabion basket (diameter 6 mm)	1 m × 1 m × 1 mm	unit	60	225	13,500.00	12,063.60	4,411.76	3,942.35

4	Wire covered by plastic (diameter 4 mm)		m	6	264	1,584.00	1,415.46	517.65	462.57

5	Employer (8 person per day for 1.5 month)		day	100	360	36,000.00	32,169.60	11,764.71	10,512.94

The total price (for 1920 m^2^)						79,285.70	70,849.70	25,910.36	23,153.50

The total price (for 0.01 km^2^)						412,946.34	369,008.85	134,949.78	120,591.13

**Table 3:** Estimation of replanted mangrove saplings of the project at Carey Island in 2008 and 2012 with consideration of inflation rate.

No.	Species of the mangrove	Price per unit (including the planting and transportation prices)	Number of mangrove	Total cost 2008 (RM)	Total cost 2013 (RM) with inflation rate	Total cost 2008 (USD)	Total cost 2013 (USD) with inflation rate
1	*Avicennia marina *	10	679	6,790.00	7,693.07	2,218.95	2,514.08
2	*Rhizophora apiculata *	10	351	3,510.00	3,976.83	1,147.06	1,299.62
Total price (for 1920 m^2^)				10,300.00	11,669.9	3,366.01	3,813.69
Total price (for 0.01 km^2^)				536,458.33	607,807.29	175,313.18	198,629.83
